# The guanine nucleotide exchange factor, *Spata13*, influences social behaviour and nocturnal activity

**DOI:** 10.1007/s00335-019-09800-9

**Published:** 2019-04-24

**Authors:** Nora Bourbia, Paige Chandler, Gemma Codner, Gareth Banks, Patrick M. Nolan

**Affiliations:** 1 MRC Harwell Institute, Harwell Campus, Didcot, Oxfordshire OX11 0RD UK; 2 Present Address: Radiation Effects Department, Centre for Radiation, Chemical and Environmental Hazards, Harwell Science Campus, Chilton, Didcot, Oxfordshire UK

## Abstract

Spermatogenesis-associated protein 13 (*Spata13*) is a guanine nucleotide exchange factor (GEF) enriched in discrete brain regions in the adult, with pronounced expression in the extended central amygdala (CeA). Loss of *Spata13*, also known as the adenomatous polyposis coli exchange factor *Asef2*, has no identifiable phenotype although it has been shown to reduce the number and size of intestinal tumours in Apc (Min/+) mice. Nevertheless, its brain-related functions have not been investigated. To pursue this, we have generated a *Spata13* knockout mouse line using CRISPR-mediated deletion of an exon containing the GTPase domain that is common to multiple isoforms. Homozygous mutants were viable and appeared normal. We subjected both male and female cohorts to a comprehensive battery of behavioural tests designed to investigate particular CeA-related functions. Here, we show that *Spata13* modulates social behaviour with homozygous mutants being subordinate to wildtype controls. Furthermore, female homozygotes show increased activity in home cages during the dark phase of the light–dark cycle. In summary, *Spata13* modulates social hierarchy in both male and female mice in addition to affecting voluntary activity in females.

## Introduction

Spermatogenesis-associated protein 13 (*Spata13*, ID 219140) codes for an eponymous protein acting as a guanine nucleotide exchange factor (GEF) for RhoA, Rac1 and Cdc42 GTPases (Kawasaki et al. 2007; Bristow et al. 2009). Notably *Spata13* is expressed in discrete loci within the brain (Lein et al. 2007) with particular enrichment in the central extended amygdala (Becker et al. 2008). Functionally, the central extended amygdala and the central amygdala have been well characterised. The regions have been shown to play important roles in anxiety-like, fear and threat (Shackman and Fox 2016; Fox and Shackman 2017), pain (Bourbia et al. 2010), feeding, reward and addiction (Koob 1999; Waraczynski 2006; Douglass et al. 2017), voluntary activity (Izumo et al. 2012) and aggressive behaviours (Haller 2018).

At the cellular level, *Spata13* has been shown to play a role in cell migration (Sagara et al. 2009; Evans et al. 2014) and glutamatergic dendritic spine formation (Evans et al. 2015). A previous study using a knock-out (KO) mouse for *Spata13* has established its importance in the development of adenomas (Kawasaki et al. 2009). However the behavioural function of *Spata13* is, as yet, unknown. A missense variant in *SPATA13* has been identified in consanguineous families with intellectual disabilities (Harripaul et al. 2018), and a genome-wide association analysis of comorbid depressive syndrome and alcohol dependence in human patients identified a single nucleotide polymorphism (SNP) in *SPATA13* amongst the top hit SNPs of the study, although only at a *p* value < 10^−5^ (Edwards et al. 2012). Because of the brain region-specific expression of *Spata13*, we were interested in investigating the neurobehavioral functions of S*pata13* using a mouse model. To facilitate such behavioural studies we generated a KO mouse of *Spata13* using CRISPR-Cas9 to delete the critical exon of isoforms containing the GTPase domain (exon ENSMUSE00000122772). These mice were then subjected to a behavioural test pipeline to investigate five critical behaviour axes: anxiety-like, nociception, working and fear-conditioned memory, social behaviour and circadian activity. From this data we demonstrate that loss of *Spata13* leads to deficits in social dominance and a female-specific elevation in home cage activity showing the importance of *Spata*13 in social hierarchy and a sex-specific effect on voluntary activity.

## Materials and methods

### Mice

All animal work was performed under the guidance issued by the Medical Research Council and Home Office Project License 30/3206, with local ethical approval. Mice were bred in the Mary Lyon Centre at the MRC Harwell Institute and, when not undergoing phenotyping, were kept in mixed-genotype groups in a 12 h light dark cycle (lights on at 07:00; lights off at 19:00) with ad libitum food and water.

### Generation and genotyping of Spata13 KO mice (SPATA13-DEL716-EM1-B6N)

*Spata13* KO animals were generated using a CRISPR-Cas9-based gene-targeting method (http://www.informatics.jax.org/allele/key/877470). Briefly, exon ENSMUSE00000122772 of *Spata13* was targeted for excision by pronuclear injection of *Cas9* mRNA and a cocktail of sgRNAs (TGCGAAACACTTCCCTACTC-TGG, GCCATATAGATTGGTGGCAG-TGG, GCTAGATGCCGGTGTAGGTT-TGG, GGCATCTAGCTTTCATGCCG-TGG) into 1-cell stage C57BL/6NTac embryos. Sequencing of F_0_ animals revealed an individual with a 716 nt deletion encompassing ENSMUSE00000122772 of *Spata13* resulting in a frameshift and premature stop codon in the following exon. Further sequencing of F_1_ progeny from this founder confirmed inheritance of the mutation. Subsequent genotyping of the colony was carried out by qPCR copy counting with a qPCR-based loss of allele Taqman assay containing primers and a FAM-labelled probe situated within the deleted region of *Spata13* (Forward Primer = CAGGGCTGTGGCTGTCTA, Reverse Primer = CTGGACGATGACGGAAACTCA, Probe = TAGTCCCTACCTGGCATTTCCTGA). A Dot1l VIC-labelled internal control was used as a reference assay (Forward Primer = GCCCCAGCACGACCATT, Reverse Primer = TAGTTGGCATCCTTATGCTTCATC, Probe = CCAGCTCTCAAGTCG). Wild type (WT) and homozygous knock out (HOM) *Spata13* mice were maintained on a C57BL/6NTac background. There was no evidence of embryonic lethality in this line.

### Behavioural testing

All behavioural tests were randomized and performed blind to the experimenter throughout the phenotyping tests. Behavioural testing was performed between 8 to 12 weeks of age with all experiments performed between 13:00 and 17:00 except for the fear-conditioning test performed between 10:00 and 12:00 during the first day of the protocol and between 10:00 and 18:00 during the second day of the protocol. Circadian analysis was performed at 24 weeks of age.

### Light dark box

A 40 cm × 40 cm × 40 cm plastic box, equally divided into dark (0 lux) and light (100 lux) compartments, was used to assess anxiety-like behaviour. The mice were placed inside the dark compartment and were able to explore both compartments through a 3 cm × 3 cm door over a 5 min period. The time spent in each compartment, the number of entries and the latency to the first entry in the light compartment were measured via video tracking using EthoVision XT software (Noldus, Wageningen, The Netherlands).

### Marble burying

The marble burying test consists of assessing how many marbles are buried by test subjects at the end of a 15-min test period. This is designed to indicate compulsive-like behaviour in mice. The mouse is placed inside a standard cage filled with the equivalent of three-cages of sawdust bedding, where 9 marbles have been placed on the bedding. After 15 min the number of unburied marbles is counted.

### Mechanical sensitivity (von Frey filament test)

Mechanical sensitivity was assessed by measuring the minimum force needed to induce a hind limb withdrawal response to a mechanical stimulus induced by calibrated monofilament applied under the foot pad. The mice were habituated to the experimental condition for 1 h on 2 days followed by testing on the third day. Mechanical stimuli were applied under the right and left foot pad of the hind limb using calibrated monofilament from 0.008 to 10 g (Touch Test™ Sensory Evaluator, Stoelting Europe, Ireland). The lowest force to induce 100% withdrawal response based on five stimuli per force was considered as the withdrawal threshold force expressed in grams (g).

### Thermal sensitivity

Thermal sensitivity was assessed using the hot plate test. Mice were placed on the hot plate (Bioseb, Chaville, France), set at 50 °C, until the first paw licking is observed. Mice were then immediately returned to their home cage. The latency to the first paw licking, expressed in seconds was considered as the withdrawal latency.

### Y maze test

The Y maze test enables the assessment of working memory using an elevated, three-arm, Y maze with a room light at 100 lux. The habituation phase consists of placing the mouse for 10 min in the start arm of the Y maze and allowing the mouse to explore two of the arms (starting and familiar arms) while the novel arm is blocked by a temporary black Plexiglas door. The mouse is then returned to its home-cage for 2 min and the temporary door is removed. The mouse is placed back in the start arm of maze and can explore freely all three arms (start, familiar and novel arms) over 5 min. The time spent and number of entries in each arm is recorded and measured using EthoVision XT. The arms corresponding to the start and familiar arms were alternated between mouse trials. Only female mice were used in this test for practical reasons related to the timing schedule of mouse experiments.

### Fear conditioning

Pavlovian threat conditioning was assessed using the Fear Conditioning System (Ugo Basile, Italy). Conditioning phase: mice are placed in a square chamber with an electric grid on the floor for 10 min. During this period, mice are exposed to a series of three conditioned stimuli (5 s tone) each coupled with a 0.5 s footshock stimulus (0.5 mA) at 150 s, 305.5 s and 461 s. Mice are then returned to their home-cage. Context phase: 24 h after conditioning, mice are placed in the same chamber and freezing behaviour recorded for 5 min in the absence of any stimuli. Mice are then returned to their home-cage. Cue phase: 4 h after the context phase, mice are placed in a round Plexiglas cylinder with vanilla fragrance spread at the top of the cylinder for 6 min during which the mouse is exposed to the same sound as in the conditioning phase. The percentage of freezing is measured using the ANYMaze video tracking system (Stoelting Europe).

### Social dominance

The social dominance tube test enables the assessment of the dominant/subordinate relationship between a pair of mice from different cages, and with similar age and weight (body weight curves up to 16 weeks available at www.mousephenotype.org). One WT and one HOM mouse are allowed to enter at opposite ends of a transparent polyvinyl chloride tube, 30 cm long and 4 cm diameter. After the mice meet at the centre of the tube, the first mouse to reverse out of the tube is considered the subordinate. Each mouse performed between 2 to 7 challenges (average of 5 challenges) against a different mouse and the percentage of challenges won was recorded.

### Circadian activity

Mice were analysed for circadian activity using the COMPASS system as described by Brown et al. 2017. Mice were individually housed under a passive infrared sensor and movement within the passive infrared field recorded in 10 s bins. Data was captured for 5 days in a 12:12 LD cycle, followed by 9 days in constant darkness. Circadian analysis was performed using custom Python scripts and excel sheets to convert activity data into AWD files for analysis on Clocklab (Actimetrics, Illinois) or Actiwatch Sleep analysis software (CamNtech, Cambridge).

### Golgi labelling and spine counts

Golgi-Cox neuronal staining was performed using the FD Rapid GolgiStain Kit (FD NeuroTechnologies Inc, USA) according to the manufacturer’s instructions. 120 µm sections of the cortex were taken using a vibratome, mounted upon charged slides, cleared in Histo-Clear (National Diagnostics, UK) and coverslipped. Neurons were viewed on a Zeiss Axio-Observer Z1 microscope and spine counts taken from at least 25 neurites per animal (n = 4 per genotype).

### Statistical analysis

GraphPad Prism 8 was used to perform the statistical analysis. For the behavioural tests, genotype and sex effects 2-way or repeated measures 2-way ANOVA were used followed by Bonferroni’s multiple comparison tests. The Mann–Whitney test was used to analyse the marble-burying test data. Repeated measures 2-way ANOVA was used for analysing activity over time in the circadian analysis. Repeated measures one-way ANOVA followed by Bonferroni’s multiple comparisons tests was used to analyse the spontaneous alternation Y maze data. Welch’s *t* test was used to compare the genotype of each sex group in the social dominance test data. For the Golgi staining data, Welch’s *t* test was used to compare the spine counts per µm between genotypes in female prefrontal cortex.

## Results

### Loss of *Spata13* does not affect memory, anxiety, nociception or spine counts

**Fig. 1:**
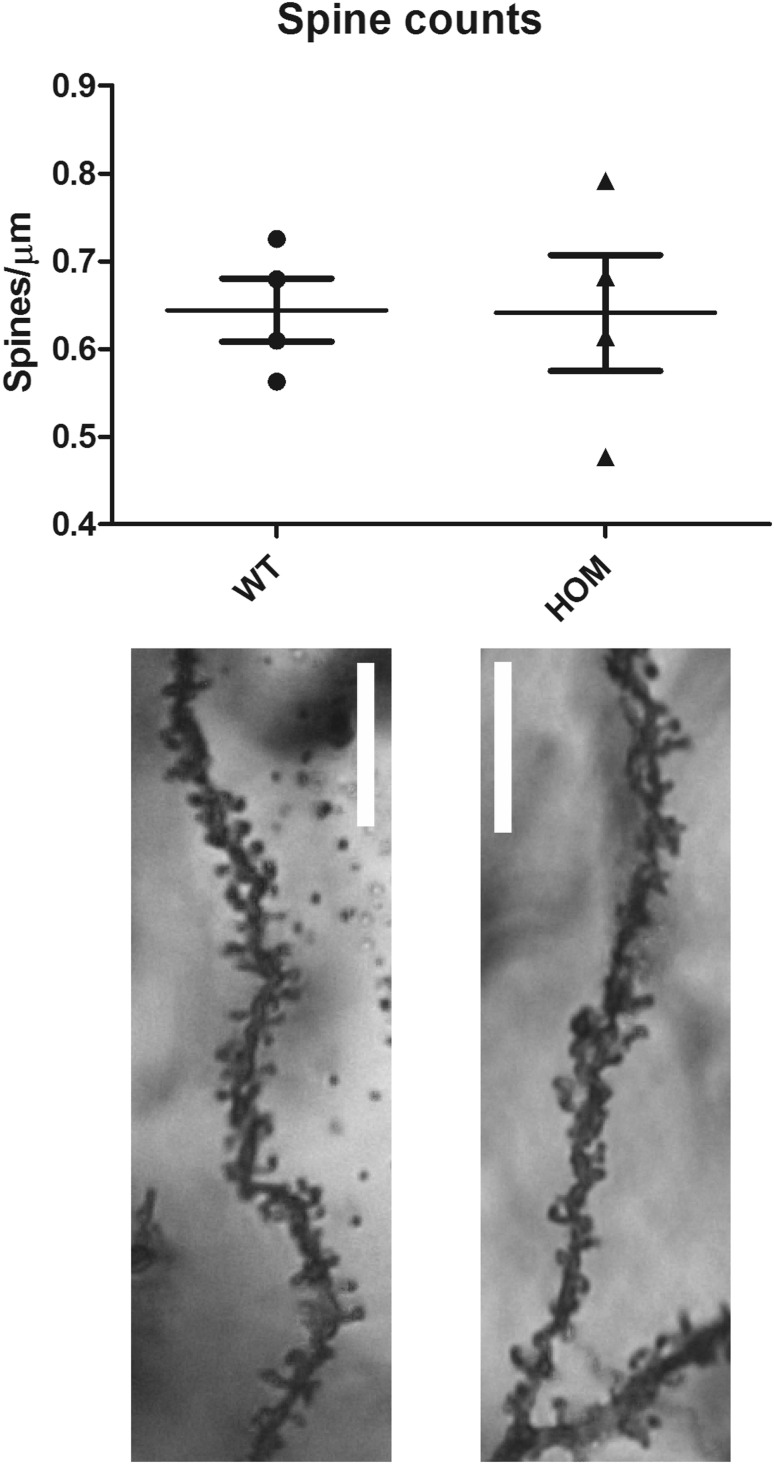
Spine counts in female prefrontal cortex. Spine counts per µm of prefrontal cortex slices do not show differences between female WT and HOM *Spata13* mice

Cohorts of *Spata13* homozygous knockout animals and wildtype controls were behaviourally assessed using the light–dark box (anxiety and exploratory behaviour), marble burying (stereotypical compulsive behaviour), von Frey filament test (mechanical sensitivity), hot plate (thermal sensitivity), working memory (forced alternation y-maze) and fear conditioning (cued and contextual memory). Analysis of the data produced in these tests found no effect of genotype in any of the parameters analysed, suggesting that loss of *Spata13* did not result in gross changes in these behaviours (Table 1). Additionally, the loss of Spata13 did not affect the number of spines per µm in the prefrontal cortex (Welch’s *t* test, *p* = 0.9667, *n* = 4 for each genotype), (Fig. 1).

**Table 1:** Behavioural measures in *Spata13* mice

Phenotyping test	Parameter measured	Statistical analysis	Significance of genotype effect
Light–dark box	Time in light	2-way ANOVA (genotype X sex)	0.3153
	No. of entries into light	2-way ANOVA (genotype X sex)	0.0676
Marble burying	Number of marbles buried by female mice	Mann–Whitney *U* test	0.7827
	Number of marbles buried by male mice	Mann–Whitney *U* test	0.8885
Mechanical sensitivity	Force to induce foot withdrawal in females	2-way ANOVA (genotype X paw)	0.721
	Force to induce foot withdrawal in males	2-way ANOVA (genotype X paw)	0.2156
Thermal sensitivity	Latency to first paw lick	2-way ANOVA (genotype X sex)	0.8203
Forced alternation y-maze	Time spent in each arm	1-way ANOVA	WT: *f* = 0.4538, *p* = 0.6210; Hom: *f* = 1.181, *p* = 0.3455
	Number of entries into novel arm	1-way ANOVA	WT: *f* = 20.04, *p* = 0.0003; Hom: *f* = 17.79, *p* = 0.0005
Fear conditioning	Contextual freezing in females	2-way ANOVA (genotype X test phase)	0.2172
	Contextual freezing in males	2-way ANOVA (genotype X test phase)	0.4395
	Cued freezing in females	2-way ANOVA (genotype X test phase)	0.5819
	Cued freezing in males	2-way ANOVA (genotype X test phase)	0.7321

No significant differences from wild-type were observed in parameters measuring anxiety, memory and nociception

### *Spata13* knockout animals show increased levels of subordinate behaviour

**Fig. 2:**
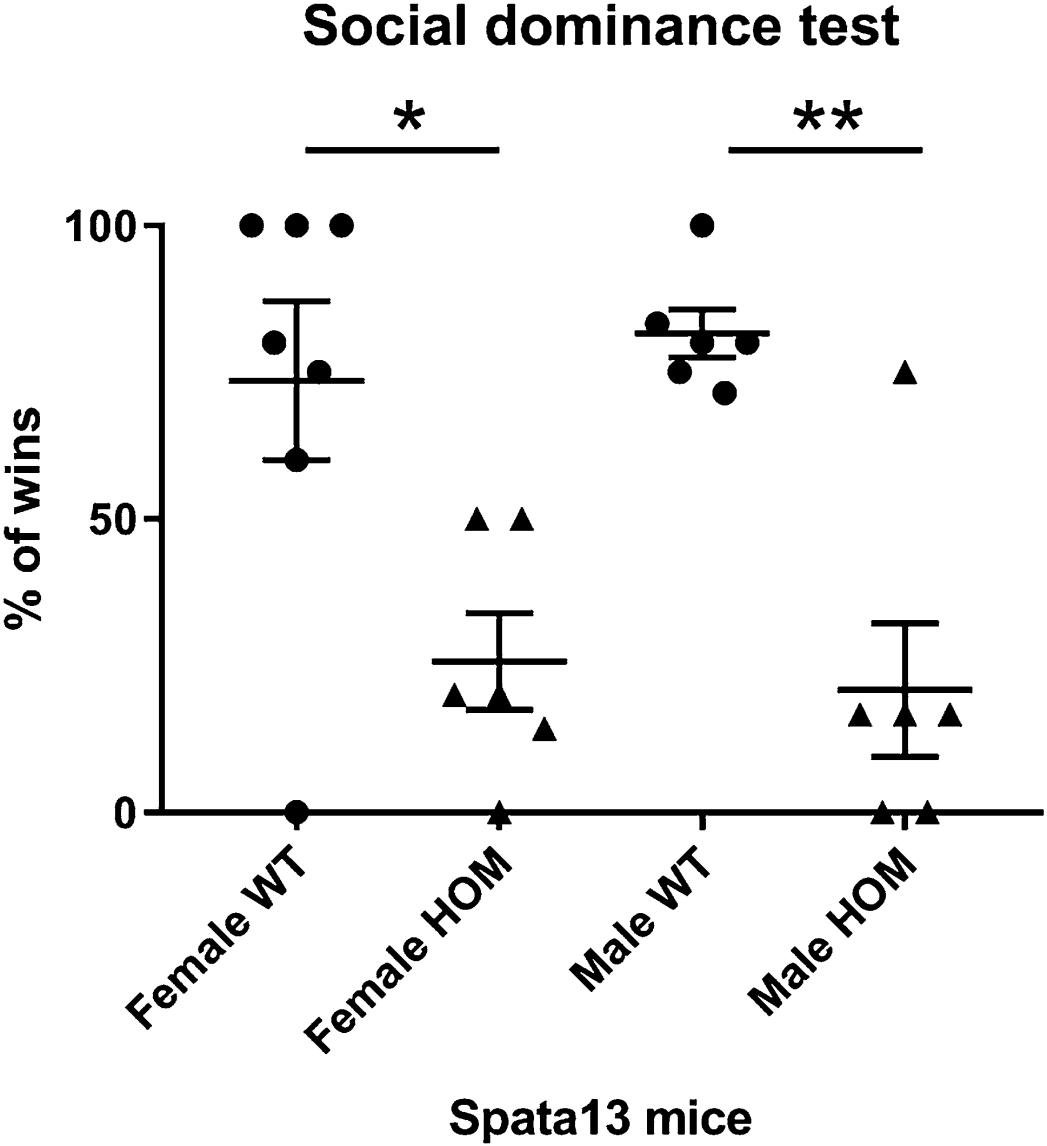
*Spata13* KO mice are subordinate to the dominant WT mice. Assessment of dominant/subordinate status using the social dominance tube test shows that female and male WT mice won more challenges when paired against HOM *Spata13* mice. *p < 0.05, **p < 0.01

The social dominance tube test reveals the dominant and subordinate mouse amongst random test pairs. Both WT female (Welch’s *t* test, *p = 0.0135, *n* = 6–7) and WT male (Welch’s *t* test, **p = 0.0021, *n* = 6) show a significantly higher percentage of challenges won compared to the HOM of the same sex indicating that mutants are significantly more subordinate in such pairings (Fig. 2).

### Female *SPATA13* knockout mice show increased activity in the dark phase of the LD cycle

Cohorts of both sexes of mice (*n* = 8 wildtype and homozygous) were analysed for changes in home cage activity and circadian rhythms using passive infrared screening. Data capture was performed for 5 days in a 12:12 light–dark cycle followed by 9 days in constant darkness. Following data analysis we found no significant differences between genotypes in any of the circadian parameters analysed (Table 2).

**Table 2:** Circadian parameters in *Spata13* homozygous mice

Parameter	Lighting conditions	Male			Female		
		Wildtype	Homozygote	*p*	Wildtype	Homozygote	*p*
Period (h)	Constant darkness	23.96 ± 0.01	23.89 ± 0.04	0.116	23.84 ± 0.03	23.81 ± 0.04	0.551
Amplitude	Light–dark	394.33 ± 44	452 ± 48	0.396	749.62 ± 93	984.25 ± 132	0.171
Amplitude	Constant darkness	269.1 ± 50	376.5 ± 50	0.258	1020 ± 125	1045.6 ± 184	0.91
Length of active phase (h)	Light–dark	12.76 ± 0.21	13.03 ± 0.19	0.361	12.98 ± 0.28	12.8 ± 0.56	0.78
Length of active phase (h)	Constant darkness	13.45 ± 0.26	13.98 ± 0.43	0.306	13.83 ± 0.3	13.49 ± 0.66	0.652
Interdaily stability	Light–dark	0.558 ± 0.02	0.562 ± 0.21	0.923	0.704 ± 0.02	0.724 ± 0.02	0.577
Interdaily stability	Constant darkness	0.407 ± 0.03	0.422 ± 0.02	0.701	0.551 ± 0.09	0.528 ± 0.12	0.629
Intradaily Variability	Light–dark	1.52 ± 0.08	1.55 ± 0.07	0.822	1 ± 0.09	1.05 ± 0.12	0.701
Intradaily Variability	Constant darkness	1.73 ± 0.09	1.72 ± 0.08	0.953	1.21 ± 0.1	1.2 ± 0.09	0.954

No significant differences between genotypes were found in any of the circadian parameters analysed in either sex

**Fig. 3:**
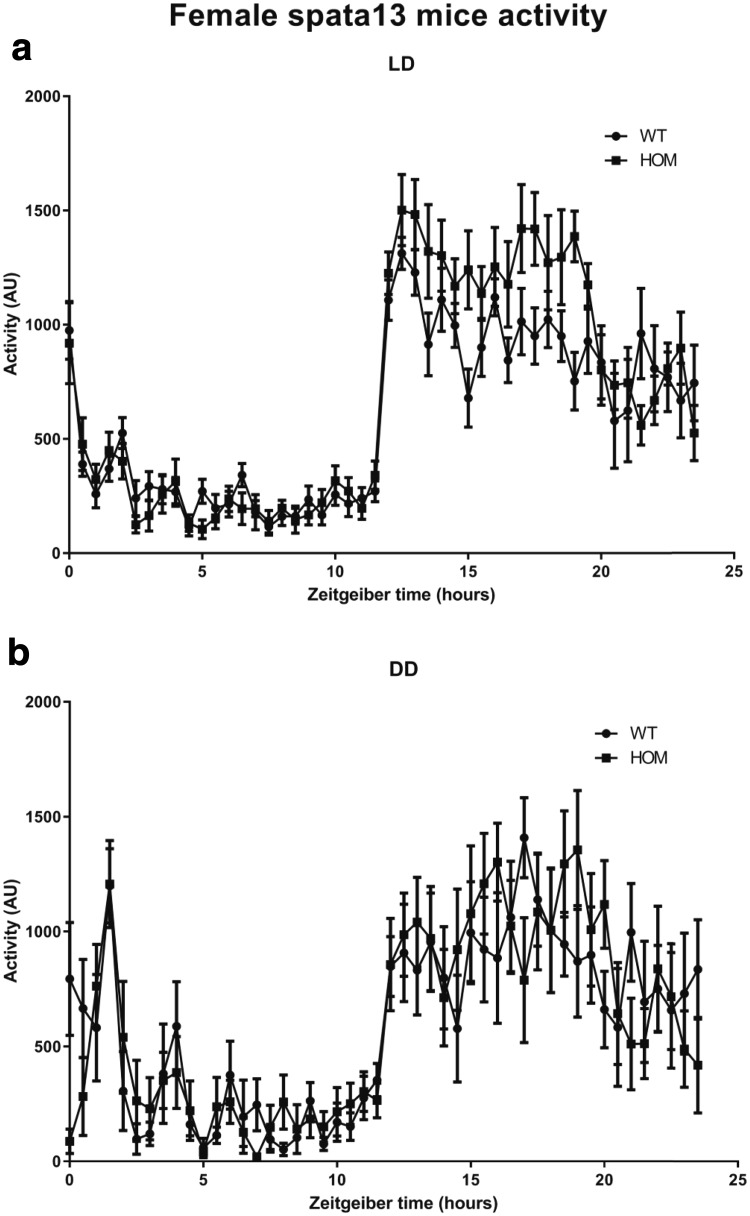
Home-cage activity in *Spata13* females. Analysis of home-cage activity over time in female animals shows that *Spata13* knockout animals show elevated activity levels in the dark phase of the light–dark cycle (**a**). No observable differences in activity were observed in female animals upon their release into constant darkness (**b**)

**Fig. 4:**
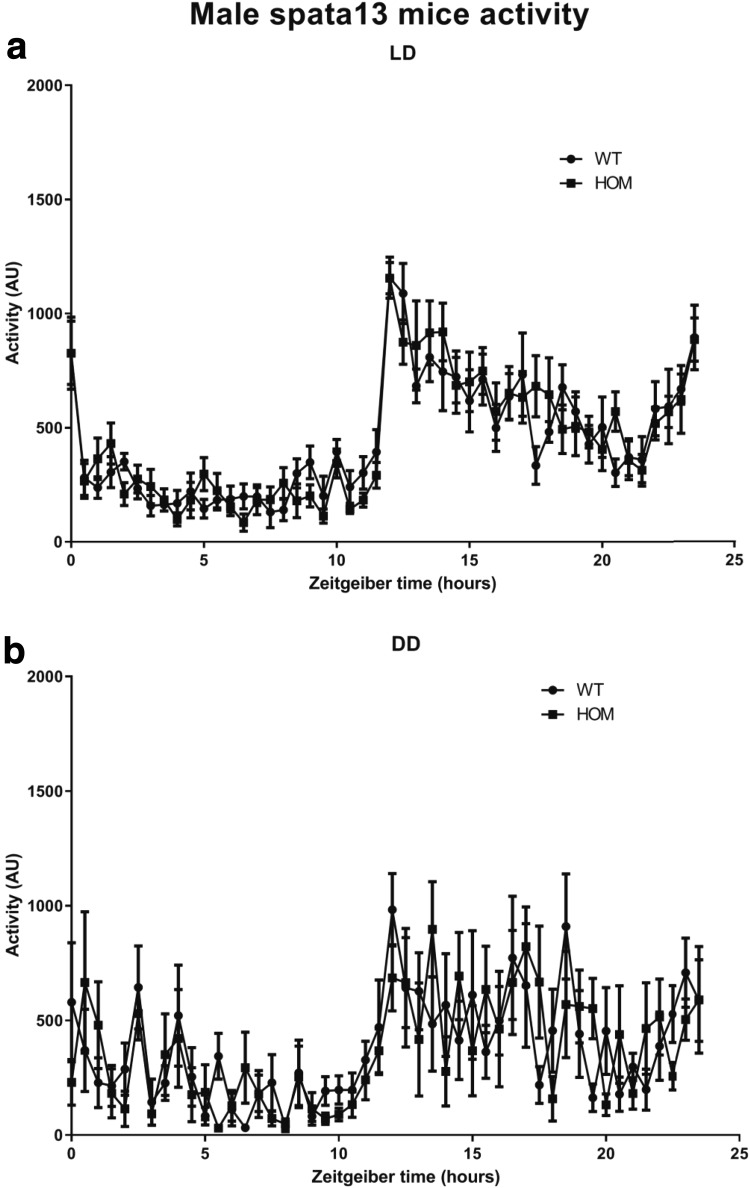
Home cage activity in *Spata13* males. Analysis of home-cage activity over time in male animals shows that *Spata13* knockout animals show no differences in activity levels in either the light–dark cycle (**a**) or upon release into constant darkness (**b**)

Activity over the 24-h cycle was analysed in 30-min bins for the female (Fig. 3) and male (Fig. 4) WT and HOM cohorts. This revealed that (Fig. 3a) female HOM *Spata13* mice show a significant elevation in activity in the first seven hours of the dark phase of the light–dark cycle compared to wildtype littermates (Female: repeated measures 2-way ANOVA, Interaction, *p* = 0.0002; Genotype difference, *p* = 0.2191; Time difference, *p* < 0.0001; Subject, *p* = 0.0104; *n* = 8). Further analysis of animal activity demonstrated that this elevation of activity was not present when animals are housed under conditions of constant darkness (Fig. 3b) (Female: repeated measures 2-way ANOVA, Interaction, *p* = 0.44; Genotype difference, *p* = 0.9143; Time difference, *p* < 0.0001; Subject, *p* = <0.0001; *n* = 8). We found no significant differences in the activity cycles of male animals (Fig. 4a). Male activity in light–dark: repeated measures 2-way ANOVA, Interaction, *p* = 0.3156; Genotype difference, *p* = 0.9572; Time difference, *p* < 0.0001; Subject, *p* = <0.0001; *n* = 8 (Fig. 4b). Male activity in constant darkness: repeated measures 2-way ANOVA, Interaction, *p* = 0.2474; Genotype difference, *p* = 0.8769; Time difference, *p* < 0.0001; Subject, *p* = <0.0001; *n* = 8).

## Discussion

SPATA13 is a Rho-family GEF expressed in specific brain regions such as the extended amygdala (Becker et al. 2008). Within the brain *Spata13* can modulate glutamatergic dendritic spine formation (Evans et al. 2015) and evidence from human patients suggests that the gene is associated with intellectual disability (Harripaul et al. 2018). These lines of evidence suggest that *Spata13* may play important roles in brain function and output and, given that behavioural data associated with the gene is lacking at present, we performed a detailed behavioural analysis of the *Spata13* knockout mouse line. Our data demonstrates that *Spata13* is implicated in social behaviour in mice of both sexes and involved in dark phase voluntary locomotion in female mice. Other behavioural aspects such as fear conditioning, anxiety-like behaviour, working memory, and thermal and mechanical sensitivity were not affected by the KO of *Spata13* despite the gene’s enriched expression in the central extended amygdala. Likewise, screening mutant cohorts in the International Mouse Phenotyping Consortium phenotyping pipeline (IMPC, www.mousephenotype.org) failed to identify parameters that were significantly affected.

A recent study combining microarray and exome sequencing of patients with non-syndromic intellectual disability (ID) identified a missense mutation in *SPATA13* in 16 patient families (Harripaul et al. 2018). While the potential mechanism by which this mutation may modulate the development of intellectual disability is unknown, it is notable that SPATA13 has been demonstrated to affect dendritic spine formation (Evans et al. 2015) and that altered spine dynamics have been associated with ID and related symptoms (Belichenko et al. 1994). However, in the present study, we found no impairments in working memory, conditioned memory or spine numbers in the prefrontal cortex in *Spata13* knockout animals. This discrepancy is most likely due to the fact that the human missense mutations may have different consequences on gene function (such as dominant negative or expression effects) than the knockout mouse line presented here. In addition, functional redundancy may also mitigate some of the phenotypes otherwise presented by the knock out. SPATA13 is a Rho family GEF which activates the small GTPases Rac, CDC42 and Rho (Kawasaki et al. 2007; Bristow et al. 2009). While GEFs play important cellular functions through the regulation of small GTPases and G proteins (Bos et al. 2007; Stanley and Thomas 2016), there are known to be at least 80 RhoGEF proteins in the human genome (Rossman et al. 2005; Goicoechea et al. 2014). It is, therefore, distinctly possible that other members of the GEF family may be able to compensate for the loss of SPATA13 in these animals.

Our data do demonstrate that *Spata13* knockout mice show subordinate behaviour when challenged by wildtype mice. Some of the neuronal networks underpinning social hierarchy are glutamatergic pathways of the medial prefrontal cortex (mPFC), which have been demonstrated to be modulated by RAS and RAP GTPases (Wang et al. 2014). Although it is not yet known whether SPATA13 functions as a GEF for RAS, a potential mechanism through which SPATA13 function may underpin social behaviours is through a reduction of RAS GTPase activation in these glutamatergic synapses. Indeed, not only is *Spata13* involved in glutamatergic dendrite spine development (Evans et al. 2015), it is also expressed in the frontal cortex of prenatal human brain (Miller et al. 2014) suggesting a possible role in the development of these glutamatergic connections. While we did not see a difference in the prefrontal cortex spine counts, the glutamatergic synapse quantity and function have not been studied. Further investigations are necessary to determine the specific function of SPATA13 within this prefrontal network and how this modulates social function.

Additionally, our study shows that female *Spata13* knockout mice have an increase in activity during the first 7 h of the active (dark) phase. Notably, the central amygdala in female rats is involved in voluntary activity during the dark phase and this control has been suggested to be modulated by ovarian hormones (Izumo et al. 2012). Chromatin immunoprecipitation has shown that estradiol recruits the estrogen receptors ERα to SPATA13 (Levy et al. 2008). While the mechanisms underlying the increase of locomotor activity of the female *Spata13* knockout mice cannot be explained here, further studies would be of merit to understand whether the link between SPATA13 and the oestrogen system inside the central amygdala could explain the observed alterations in activity. It is also interesting to note that we did not observe any hyperactivity in the behavioural tests presented here, all of which were performed during the light phase. It would, therefore, be worth further testing the female mice for hyperactivity during the dark phase and whether this is associated with any additional behavioural anomalies.

## Conclusion

Here, we have shown that knocking out *Spata13* has no effect on viable embryonic development, circadian rhythms, learning and fear conditioning and anxiety-like behaviour. However, *Spata13* is involved in the mechanism of social hierarchy in both female and male mice, and in nocturnal activity in female mice.
